# Motivating tones to enhance education: The effects of vocal awareness on teachers' voices

**DOI:** 10.1111/bjep.12737

**Published:** 2025-01-11

**Authors:** Silke Paulmann, Netta Weinstein

**Affiliations:** ^1^ University of Essex Colchester UK; ^2^ University of Reading Reading UK

**Keywords:** motivational tone of voice, prosody, SDT, voice training

## Abstract

**Background:**

Effective classroom communication is key to shaping the learning environment and inspiring student engagement. And, it's not just what is said, but how it's said, that influences students. Yet, few (current or future) teachers receive education on vocal pedagogy.

**Aims:**

This study examined the impact of raising vocal awareness in teachers on their voice production through delivering a voice training program.

**Method:**

Specifically, we explored how primary school teacher trainees produced motivational (either soft, warm, and encouraging, or harsh, pressuring, and controlling) and neutral communications before and after the delivery of a voice education program that concentrated on raising voice awareness, vocal anatomy, exercise techniques (e.g. breath control, voice modulation), and voice care. Hypotheses: We hypothesised that trainees' voice production would change over the course of the program and lead to more ‘prototypical’ displays of motivational prosody (e.g. softly spoken encouraging intentions vs. harshly spoken controlling intentions).

**Results:**

Results indicated a noticeable difference when communicating motivational intentions between pre‐ and post‐training voice samples: post training, trainees spoke more slowly and with reduced vocal effort irrespective of motivational intention, suggesting that raising vocal awareness can alter classroom communications.

**Conclusion:**

The results underscore the importance of vocal awareness training to create a supportive and autonomy‐enhancing learning environment.

## INTRODUCTION

A substantial literature concludes what may be apparent to some: that teachers benefit students' motivation, participation, and well‐being when they foster a supportive and engaging classroom atmosphere (e.g. review in Guay, 2022). While recent empirical evidence on how motivational voice use in the classroom can help create this atmosphere is still in its infancy, past work (Paulmann & Weinstein, 2023) suggests that the extent that teachers use their tones of voice to motivate through supporting the sense of choice and interest of children (i.e. to speak in an *autonomy‐supportive* way) or by conveying pressure and demands (i.e. to speak in a *controlling* way) is important for children's well‐being, self‐disclosure to teachers, and cooperation with teachers' expectations.

Moreover, many years of research on voice education in teacher training collectively highlight the importance of vocal awareness and at the same time the lack of systematic training available for trainees (e.g. Kovacic, 2005; Nusseck et al., 2021; Roy et al., 2004; Velsvik Bele, 2008). Surprisingly, the effects of vocal awareness training on the production of different motivational teacher voices (here: autonomy‐supportive and controlling) have not been tested. However, raising awareness around the effects of voice in the classroom and on vocal pedagogy more generally should play a contributing role given the prominent role voice plays in a teacher's toolkit to convey information and shape pupil‐teacher relationships. The current study aimed to address this gap in the literature by testing the impact of a voice education program on teachers' production of motivational intentions, which were assessed through acoustic measurements of voice taken before and after the vocal awareness program to specify the impact of voice training on pitch, loudness, speech rate, and voice quality.

### The effects of voice training in teaching

Various attempts to train professionals' voices have been made, with the broad goals of improving the effectiveness of communications. A primary interest has been to investigate whether vocal quality improves and/or voice disorders disappear or can be prevented. Such training can be extensive (for example, 8 months of weekly coaching) (Leino & Kärkkäinen, 1995; as cited in Velsvik Bele, 2008) or brief, lasting just 6 h (Timmermans et al., 2011). Training has predominantly been tested with primary school trainees or teachers of children aged 5–11 (Kovacic, 2005; Nusseck et al., 2021; Roy et al., 2004), secondary school teachers of children aged 11–16 (Nusseck et al., 2021; Roy et al., 2004), and teachers within schools for children with special needs (Nusseck et al., 2021). The success of voice education programs is usually evaluated in laboratory studies where teachers provide voice samples outside the school context (e.g. sustained vowel production).

Interestingly, even during brief training (e.g. 6 h in a large group setting), acoustic results suggested that students who received voice training alter their vocal behaviour between training sessions. For example, Timmermans and colleague's (2011) study found that students receiving brief training expanded their voice range and showed changes in the way they were able to control pitch (e.g. maximum pitch produced) and loudness (e.g. lowest amplitude produced). Specifically, female participants showed changes related to maximum pitch and men related to minimum intensity. Combined, these results suggest that even short voice training programs can have an effect on vocal production.

The majority of published training programs to date have focused on voice health factors, with the goal of the training program ensuring that vocal behaviours displayed in the classroom do not lead to vocal dysfunction behaviours (e.g. continuous straining of voice). While this goal is important in its own right, many existing program evaluations do not offer guidance to teachers in terms of which voices to use to create supportive learning environments.

### Autonomy‐supportive versus controlling motivational tones

Considering how voices shape supportive learning environments is crucial when thinking about the power of voice in the classroom. A wealth of literature highlights the benefits of autonomy‐supportive versus controlling learning environments. Embedded in self‐determination theory (SDT), (Deci & Ryan, 2000), much work in educational psychology points to the benefits of creating autonomy‐supportive teaching practices such as providing young people with options and avoiding punishments and pressures where possible (see, for example, Reeve & Shin, 2020, for overview). Specifically, it has been argued that a teacher's motivating style can have profound effects on students' wellbeing and educational outcomes. Of interest is whether teachers convey that they support the pupils' autonomy, or sense of agency and choice as students engage the learning process, or conversely, whether teachers convey control through pressuring and demanding learning be pursued in particular ways (e.g. Reeve, 2009).

Importantly, autonomy‐supportive versus controlling teaching can be communicated through teachers' spoken content, and much attention to how the words that teachers use affect students' learning experiences and outcomes (Baker & Goodboy, 2019; İhtiyaroğlu, 2019). Little attention within education has been given to addressing these motivational tones, but Reeve and Cheon et al. (2020) argue that teachers who support autonomy in students display curiosity and openness and when students display problems, teachers can adopt an ‘interpersonal tone’ that demonstrates that teachers are paying attention to their students.

In recent lab‐based experimental work, when young people hear teachers' controlling versus autonomy‐supportive tones of voices as they convey the same educational messages, they anticipated reduced well‐being and were less willing to disclose to teachers. Results also revealed that listening to autonomy‐supportive sounding voices increased pupil's autonomy and relatedness need satisfactions (their sense of being choiceful and supported in self‐expression, and their sense of closeness with teachers) and also indirectly increased their anticipated well‐being, intention to cooperate and intention to self‐disclose (Paulmann & Weinstein, 2023). Of relevance here is the idea that the ‘interpersonal tone’ can be achieved by using invitational language, displayed not only through *what* teachers are saying, but also *how* they are saying it. Returning to the broader literature on motivating students, adopting such teaching practices can help students' motivation and increase their academic functioning including by fostering initiative (Reeve & Shin, 2020) and classroom engagement or academic achievement (Cheon et al., 2016).

### Motivational prosody

Work conducted outside of the domain of education also underlines the importance of modulating tones of voice to convey autonomy‐supportive versus controlling motivational qualities. For example, Weinstein et al. (2018) initially asked students from the US and the UK to speak motivationally laden sentences (e.g. ‘you have to do it my way’ (controlling message) or ‘you may do this if you choose’ (autonomy‐supportive message)) while they were recorded but with no instructions on how to speak the materials. Sentences with motivationally different content were distinguished at the vocal level: autonomy‐supportive content was spoken with lower pitch, lower intensity, slower speech rate and less harshness in students' voices when compared to content that conveyed controlling or pressuring content.

In a subsequent study, trained speakers were provided with information around what an autonomy‐supportive (as opposed to controlling) speaking style aims to express (e.g. signal a sense of choice to the listener; provide support; show an interest versus trying to enforce an opinion; exert pressure; lack interest in other person's view). Speakers were then provided with materials that could be spoken in either context (e.g. ‘It is time to go to school now’). Similar to the first study, autonomy support and control intentions were expressed differently, with autonomy support spoken less loudly (i.e. reduced *amplitude*), less forceful (i.e. less energy in high frequency bands of the vocal signals), and more slowly (i.e. lower *pace*). The only acoustic measure that was not comparable to student exemplars was fundamental frequency (f0, perceived as pitch; Weinstein et al., 2018), suggesting that mean pitch (i.e. how high or low someone speaks on average) is not a driving force when communicating motivations through voice cues. Since these original studies, additional support for the acoustic distinction between different motivational intentions has been obtained in different contexts (parenting, education; see for example, Paulmann & Weinstein, 2023; Weinstein et al., 2019) and for languages other than English (Paulmann et al., 2018; Vrijders et al., 2024). Collectively, these studies highlight that different acoustic parameters work together to convey the motivational intention of a speaker, similar to what has been proposed in the emotional prosody literature (e.g. Banse & Scherer, 1996; Paulmann et al., 2008), which has characterized acoustic profiles for different basic emotions. Crucially, past work highlights that it matters to listeners whether a speaker uses a soft or harsh sounding voice (a.k.a. *voice quality*). In other words, the way a speaker sounds (soft or harsh) when motivating others, especially when trying to enforce control, is of particular importance. While mean pitch levels were previously not found to be predictive of motivational intention, other pitch related measurements such as vocal range (wider range indicates more modulation) have not yet been extensively studied.

### Current study

To the best of our knowledge, no empirical attention has been given to understanding whether or how voice training can impact teachers' *motivational* voice production. Given the potential importance of such training on the student experience (Paulmann & Weinstein, 2023), teacher training may be an important resource to improve teacher‐student communication. Thus, we set out to investigate how the delivery of a voice education program that concentrates on delivering information around vocal anatomy, exercise techniques (e.g. breath control, voice modulation), voice care and motivational voice use (what type of voices to use in which situation) affects teacher trainees' communications. Specifically, we focused on the production of encouraging (i.e. autonomy‐supportive sounding) as well as pressuring and controlling sounding voices and explored how those vocal productions changed through a voice training program. Following views that autonomy‐supportive teaching practices can be learned and that instructing teachers on how to achieve this communication can benefit the classroom environment (Reeve & Shin, 2020), we tested the effects of raising awareness around the benefits of invitational and open non‐verbal language use in the classroom on voice production of teachers.

We hypothesised that educating trainees about the benefits of encouraging sounding voices and the negative impact of pressuring or controlling sounding voices would affect the way these communications are produced before and after this training occurred. Based on past voice training reports (e.g. Timmermans et al., 2011; Velsvik Bele, 2008), we anticipated that effects of voice training should be most prominent when looking at changes for speech rate (slower and more articulate) and vocal quality (e.g. reduced harshness, strain). We specifically anticipated that, autonomy‐supportive sounding voices would employ a softer voice quality and be spoken more slowly than controlling or pressuring sounding voices and that this effect should be amplified after training. It remains to be seen in how far the voice training also affects the production of controlling voices, which often display harsh voice quality (i.e. it is unclear if pressuring instructions are also spoken with a softer sounding voice after the training given that vocal quality changes have been observed previously).

## METHOD

### Participants

Trainee students who had recently started their first year of working as school teachers and were supported through the same student‐centred teacher training (SCITT) organization (Essex/Thames) were invited to participate.^1^ All trainees were working in primary schools in Essex/Thames (UK), teaching children between the ages of 4 years and 11 years. Teacher trainees who participated in both recording sessions (which ran in November 2021 and January 2022; see below) were included in statistical analysis (in total, 30 teacher trainees were tested, 23 of them were women; Age range between 22 and 53 years old^2^). Sensitivity analysis indicates that with this sample size we were able to detect moderate effect sizes of *d* = .50 at power = .80 and assuming *α* = .05.

### Voice training

The voice training delivered was part of SCITT Essex/Thames standard program during teacher training and delivered through a professional teacher voice training company who employs voice coaches (including one fully qualified speech and language therapist) and follows a set curriculum over four training sessions. The program followed recommendations in the literature (Kovacic, 2005) to include components on (1) voice awareness (e.g. informing trainees about the benefits of using supportive voices, what research reveals about using harsh voices, introducing a voice map that helps to know which voice to use for which situation); (2) vocal anatomy (e.g. introduce vocal production mechanisms; important terminology); (3) vocal hygiene (e.g. how not to strain the voice; breathing techniques); and (4) voice care (e.g. why hydration is important; avoid spicy foods). Of specific interest to the current study is the voice awareness component as the provider offers a program that focuses on five teaching voices, namely the advisory/comforting voice, encouraging voice, centred neutral voice, firm voice and extra firm voice.

In Training Session 1, the program provider introduced the different teaching voices and linked them to specific contexts (e.g. discussions revolved around the importance of an encouraging voice for providing support, for praise and compliments, and for encouragement of good behaviour while sharing that a firm voice may be used for demands/warning after making requests and achieving silence if not managed with centred neutral voices). Trainees were told that encouraging and comforting voices require a warmer tone colour while firm voices require more power and firmer tone colour. Session 1 also provides information on voice effects on children, both in terms of behavioural outcomes and neural processing as shown in past studies (e.g. Paulmann & Weinstein, 2023; Paulmann et al., 2019). Specific emphasis was given on highlighting that firm and extra firm voices might lead to stress‐like responses in children if used extensively and in the wrong contexts (e.g. as a standard tool for behaviour management).

In Session 2, trainees were provided with an understanding on vocal anatomy and voice production mechanisms. They were introduced to the basic vocal techniques of postural alignment, breath support, resonance and articulation. They were also provided with information around vocal hygiene and taught ways they can look after their voice. Trainees were provided with ideas on how to warm up and train their voice, what to do and what not to do in terms of voice care. In Sessions 3 and 4, trainees were given the opportunity to practise vocal delivery skills, including knowledge around the effects of pace, pitch, rhythm, intonation and inflection. Trainees were first provided with an understanding of how to master a centred neutral voice that can be used when not directly motivating students but communicating information (‘This term we will work on our times tables’), before the vocal delivery skills were linked to an expanded vocal delivery map (comforting, supporting, firm, extra firm voice). Finding and staying within the centred‐neutral voice was practised (e.g. by humming the song ‘Happy Birthday’). Trainees were also reminded that children can feel uncomfortable when trainees speak in a high pitched voice. This is important as vocal coaches shared that trainees often have the tendency to speak in a high pitched voice when addressing primary school children. Both, individual (from trained speech and language therapist/vocal coach) as well as peer group support was provided. See Table 1 for a summary of these sessions.

**TABLE 1:** Short outline of session focus and content provided by the teacher training company.

Session	Focus	Content
1	Voice awareness	Trainees receive information about the effects of voice on children; voice map is introduced (which voices should be used in which situation).
2	Vocal anatomy, vocal hygiene and vocal care	Trainees learn about key terminology, vocal production mechanisms, postural alignment, breath support, resonance, articulation, faulty vocal habits, and voice protection techniques.
3	Revision and practice	Trainees practise vocal delivery skills (e.g. pace, pitch, intonation, rhythm). Centred neutral voice is practised. Vocal delivery map applied.
4	Revision and practice	Mentor work revisited, share how different voices were used in the classroom, practise of vocal delivery skills.

### Teacher voice recordings

Voice recordings from trainees were obtained at two time points, one at the start of the voice training (Training Session 1, or Time Point 1) and once at the end of the voice training (Training Session 4, or Time Point 2). Voice recordings were provided on a voluntary basis and we asked teacher trainees for their consent to analyse voice files on the recording website. We invited all attendees of the training to go to a specifically constructed online recording website (due to COVID‐19 restrictions) which meant that experimenters lacked control over exact positioning and quality of recording equipment. It should be noted that the audio recorder does not allow recording from phones and all trainees were instructed to use their laptops (or desktops) and the microphones (either built‐in or external) that they used for online meetings. Past work exploring the influences of recording environments has identified that some parameters are more affected by recording differences than others (e.g. Van der Woerd et al., 2020).The recording site asked trainees for their participant number (previously provided to them through one of the instructors) and gender and age before asking trainees to speak out 15 sentences displayed on screen in a way that felt natural to them (i.e. no instructions on which tone of voice to use were provided in either recording session). Prior to the training session, we created 15 example sentences that could be distinguished in terms of content either biasing towards disciplinary actions’ (e.g. ‘This is your last warning!’) or supportive and encouraging actions (e.g. ‘Well done on that task!’). There were also sentences that were considered ‘neutral’ in that they didn't bias towards a specific dimension and the provider would use as examples for the ‘centred neutral voice’ (e.g. ‘We are going out now.’). Teachers received no instructions around potential domains/usage from our recording website.

### Acoustic analysis

Acoustic analyses were carried out with praat (Boersma & Weenink, 2023) using customized scripts that automatically extracted a range of acoustic parameters for each utterance provided by each speaker (fundamental frequency (f0), amplitude, duration). The pitch range for men was set at 75 Hz–450 Hz and for women at 125 Hz to 650 Hz. Voice quality as measured with cepstral peak prominence (CPP) was extracted using VoiceLab (Feinberg & Cook, 2020). We concentrated on measurements related to pitch (mean f0, f0 range), loudness (amplitude range), duration (seconds) and vocal quality (CPP) to explore the effects of voice awareness training on vocal production because these variables play a prominent role in the past motivational and emotional prosody literature (e.g. Paulmann et al., 2008; Weinstein et al., 2018). Specifically, we extracted the measures that were previously reported in Paulmann et al. (2019) and Weinstein et al. (2018). Specific parameters diverged slightly from past work given the online recording session during COVID‐19 that limited control of recording equipment which would most prominently affect loudness/energy measurements. To assess whether recordings were affected by lack of control over recording sites and equipment, we calculated Signal‐to‐Noise ratios (SNR) with Matlab, using 1 s of silence (i.e. non‐speech signal) from the original recording as our measurement for background noise. Recordings at time point 1 had an average SNR of 50 dB compared to recordings from time point 2, where the SNR was calculated at 45 dB. Table 2 lists means for each acoustic variable for both pre‐ and post‐training conditions.

**TABLE 2:** The table lists acoustic variables of interest. Values are mean values averaged across participants for pre‐ and post‐training conditions.

Acoustic measure	Encouraging		Firm		Neutral	
	Pre	Post	Pre	Post	Pre	Post
f0 (Hz)	221.3	226.7	209.3	206.0	198.7	201.2
f0 range (octaves)	1.0	1.1	.9	1.0.	1.1	1.2
Duration (seconds)	1.6	2.0	1.4	1.8	2.7	3.1
amplitude range (dB)	54.5	58.2	54.5	58.5	56.1	58.6
CPP (dB)	27.7	27.9	27.0	28.1	29.6	30.0

Abbreviations: CPP, cepstral peak prominence; f0, fundamental frequency.

### Analytic strategy

Acoustical measurements (f0 [Hz], pitch range [interval measured in octaves], duration [seconds], amplitude range [dB], CPP [dB]) were entered into a general linear model (GLM) with the repeated‐measures variables Motivation (with three levels [supportive, pressuring, neutral]) and Time Point (two levels [before training session, after training session]).

## RESULTS

### Mean f0

The main effect of Time Point was not significant, *F*(1, 29) = .133, *p* = .718, *η*_*p*_^2^ = .005, suggesting that overall mean pitch used by trainees did not differ between training Session 1 and Session 4. However, Motivation predicted pitch, *F*(1, 29) = 31.62, *p* < .001, *η*_*p*_^2^ = .522, suggesting that teachers varied their pitch depending on the motivational content of sentences spoken. Pairwise comparisons identified that sentences requiring a supportive voice were spoken with a higher pitch than sentences requiring a neutral (*p* < .001) or pressuring (*p* < .001) voice. The latter two also differentiated from each other (*p* = .003) with centred neutral voices employing the lowest pitch. The Time Point × Motivation interaction was not significant, *F*(2, 58) = 1.72, *p* = .189, *η*_*p*_^2^ = .056, indicating that mean pitch used did not differ for conditions before and after trainees participated in the awareness training.

### Range f0

The main effect of Time Point was significant, *F*(1, 29) = 8.37, *p* = .007, *η*_*p*_^2^ = .224, revealing that teachers increased their pitch range after the training. There was a significant main effect of Motivation, *F*(1, 29) = 34.29, *p* < .001, *η*_*p*_^2^ = .542, such that neutral sentence content led teachers to use a larger pitch range when compared to content that required a supportive sounding voice (*p* = .008) or firm voice (*p* < .001). When speaking in a pressuring way, teachers also restricted their range compared to talking in a supportive voice (*p* < .001). The Time Point × Motivation interaction was not significant, *F*(2, 58) = 1.56, *p* = .220, *η*_*p*_^2^ = .051.

### Duration

The main effect of Time Point was significant, *F*(1, 29) = 53.73, *p* < .001, *η*_*p*_^2^ = .649, indicating that teachers spoke more slowly after participating in the training sessions. There was also a significant main effect of Motivation, *F*(1, 29) = 482.59, *p* < .001, *η*_*p*_^2^ = .943, such that neutral sentence content was spoken most slowly compared to content that required a supportive (*p* < .001) or pressuring (*p* < .001) voice. The content benefitting from a pressuring voice was spoken faster (*p* < .001) than that requiring a supportive voice. The Time Point × Motivation interaction was not significant, *F*(2, 58) = 1.73, *p* = .842, *η*_*p*_^2^ = .006.

### Amplitude range

There was only a main effect of Time Point, *F*(1, 29) = 5.81, *p* = .022, *η*_*p*_^2^ = .167, revealing a wider range of loudness use after training. The effects of Motivation, *F*(1, 29) = 2.390, *p* = .101, *η*_*p*_^2^ = .076, and Time Point × Motivation, *F*(2, 58) = 1.99, *p* = .146, *η*_*p*_^2^ = .064, were not significant.

### CPP

The main effect of Time Point revealed significant differences for CPP measures taken before and after training, *F*(1, 29) = 11.59, *p* = .002, *η*_*p*_^2^ = .285, indicating that teachers' voices were smoother after training. There was also a significant main effect of Motivation, *F*(1, 29) = 75.37, *p* < .001, *η*_*p*_^2^ = .722. The planned pairwise comparisons revealed that neutral sentence content was spoken softer than content intended to be spoken in a supportive (*p* < .001) or firm (*p* < .001) voice. The Time Point × Motivation interaction was also significant, *F*(2, 58) = 3.41, *p* = .04, *η*_*p*_^2^ = .105. Follow‐up pairwise comparisons revealed that there were only significant differences in voice quality for content meant to be spoken in a pressuring voice when comparing before and after training vocal production (*p* < .001). The version produced after training was spoken less harshly than the version produced before the training.

Taken together, results revealed differences in acoustic production before and after voice awareness training took place. Specifically, teacher trainees spoke with a larger pitch and loudness range, at a slower pace and a softer sounding voice after training completion.

## DISCUSSION

This study set out to test the effects of voice awareness training on vocal production for motivational intentions in hierarchical relationships—those in which teachers speak to their students. Results indicated that voice awareness training affects the ways that teachers modulate their voice by increasing their pitch and loudness range, and the speed with which they speak, namely to speak more slowly after voice training. Teachers also display an overall smoother voice quality after the training. These effects were demonstrated irrespective of motivational intention expressed, suggesting that voice awareness training leads trainees to employ a more modulated voice, slower pace and a softer tone for all their teaching, regardless of the content expressed. These findings are well aligned with past work that demonstrated that even short vocal training programs can impact vocal production of teachers (e.g. Timmermans et al., 2011).

### Motivational prosody

Past work has tested how different speakers adapt the way they speak depending on motivational intentions (e.g. Paulmann et al., 2018; Weinstein et al., 2018). Consistently, this work has highlighted that speakers expressing pressure and control through voice cues use a louder voice, faster speech rate, and harsher sounding voice than when they intend to express autonomy support. These patterns of speech have been reported for vocalised messages directed at adults as well as children (in the parenting context) using both experimental and naturalistic research designs (see Paulmann et al., 2018). There has also been an attempt to compare speech from a general sample versus actors expressing motivational intentions. The latter, who are considered professional voice users (not unlike teachers) used pitch differently to lay speakers. In the case of professionals, autonomy support is expressed with a higher mean pitch when compared to controlling messages. The opposite was found for lay speakers (Paulmann et al., 2018). Current results extend these findings into the teaching context. Similar to actors, teachers in the current study used a higher pitch when communicating support and encouragement and they also kept their range more restricted than when speaking in a neutral way (i.e. they stayed high pitched throughout the sentence).

Past work has associated power with positive emotions (e.g. Keltner et al., 2003), which are, in turn, associated with an increase in pitch (e.g. Banse & Scherer, 1996). Some have linked the two concepts to say that those high in power therefore speak with an increased pitch when trying to demonstrate their power (e.g. Ko et al., 2015). Teacher‐pupil relationships are hierarchical in nature and it could be argued that teachers who are speaking more positively will ultimately be perceived as more ‘powerful’, or ‘influential’ potentially aiding with classroom discipline without the need to use controlling or pressuring language. Indeed, low pitch use has been associated with trying to sound dominant (Tusing & Dillard, 2000) and the voice awareness training provided here seems to help teachers to move away from trying to dominate students in their teaching.

We could not examine mean loudness in the present context given the lack of control over baseline measurements controlling for this parameter (specifically, because teachers could have been at different distances from their microphones), but the range of loudness (maximum—minimum loudness produced) did not vary across different motivational intentions. Interestingly, the previously identified effect of supportive statements being spoken with a significantly smoother and softer sounding voice than controlling sentences was not found here (though the means point in the correct direction). Neutral voices were spoken most mildly. Note, however, that the voice quality index we used in the present work differs from the one used in the past given our technical constraints using an online recording set up during the COVID‐19 pandemic. Overall, results are similar to observations from the past (e.g. Weinstein et al., 2018) and indicate that different motivational intentions are expressed with different voice patterns, even when speakers are not provided with guidance on ‘how a voice should sound’.

### How voice awareness training changes the way speakers express motivational intentions

Traditionally, voice training has been linked to the goal of improving vocal health of teachers as it raises awareness around vocal hygiene and tends to convey best practice (e.g. drink plenty, avoid vocal abuse). Voice quality measures have been of particular interest in the past to help teachers avoid hoarse or raspy sounding voices (Hazlett et al., 2011). The present findings align with the view that voice awareness training leads to smoother sounding voices. Specifically, through strengthening vocal support, trainees are less likely to fall back on phonation types that diverge from modal production (e.g. breathy, tense). However, the vocal awareness training given to trainees here expands on traditional programs that focus on vocal health only: teacher‐trainees were provided with information about the benefits of using encouraging and supportive voices and the pitfalls of extensively relying on controlling or pressuring sounding voices. Thus, the awareness training focused on voice as a didactic tool with the goal to improve trainees' mastery of vocal abilities. Within the sessions, trainees are encouraged to try different approaches in the classroom, with clear guidance to stay away from firmer tone colours (https://the5voices.com/the‐5‐voices/resources/) for the majority of classroom interactions (e.g. conveying instructions, expectations for behaviour, requests).

While results highlight that trainees' vocal expressions for the different motivational content differed already at the beginning of the training (e.g. pressuring content was spoken more firmly than autonomy‐supportive sentences; different pitches for different motivational intentions), the trainees' vocal approach was further modified *after* training sessions had taken place. For example, even when expressing controlling or pressuring sentence content (e.g. ‘I've told you not to do that!’), teachers employed a softer sounding way of speaking after the training, suggesting they tried to tone down the harshness in their voice. A larger pitch range used after training further implies that the way trainees' speak has been altered through the training and that the training predominantly leads to shifts towards more positive vocal expressions as larger variability in pitch has been associated with producing positive emotions (e.g. Kamiloğlu et al., 2020). Finally, slowed speech after training might imply that teachers pay more attention to their articulation as reduced pace helps to control speech output (Yorkston et al., 1990).

### Potential benefits of observed vocal changes

There is a wealth of evidence that *what* is said in classrooms has an effect on pupils' motivation and behaviour. For example, fear‐focused messages can lead to feelings of threat, increase anxiety, and reduce motivations (e.g. Putwain & Best, 2011). Moreover, vocal delivery research has focused on studying how voice use can support learning and memory. Teachers who do not vary their tone struggle to keep pupils' attention (Schmidt et al., 1998), pitch variation can improve listeners' recall of items (Rockwell, 1996), and better voice quality helps with understanding speech (Imhof et al., 2014). Recommendations for teachers having vocal awareness—an understanding and control of their voices in the classroom—are not new (c.f. Evans and Savage, 2017) and the Teaching Agency has previously recommended that ‘trainees should be able to vary the tone and volume of their voice to teach effectively and manage behaviour’. Yet, surprisingly few training programs seem to fall back on empirical evidence to highlight why employing specific voices in specific contexts can be beneficial to children. Here, we show that voice awareness training that includes both information on vocal hygiene/anatomy and around how different voices can affect children can lead to changes in how voice is employed in different contexts.

Past work has often linked a loud voice to authority, including suggestions that loud voices can help with classroom management (e.g. Kovacic, 2005). The current findings suggests that raising awareness about the impacts of firm voice use on children's well‐being, self‐esteem and willingness to disclose problems to teachers (Paulmann & Weinstein, 2023) can lead to teachers employing a less stern voice even when producing materials that signal the need to comply (e.g. ‘I need your attention!’, ‘I've told you not to do that!’). The observed increase in pitch range used by trainees after awareness training also provides support for the idea that all materials were produced with the intention to sound ‘positive’ (e.g. Kamiloğlu et al., 2020). Adjacent work from the mating literature has shown that an increase in pitch variability is linked to expressing an interest in a partner (see Pisanski et al., 2018). Extending this work into the classroom, the combined observed vocal changes suggest that productions *after* training generally sound more positive (e.g. friendly, approachable) and soft (linked to encouraging or supportive communications). Collectively, the findings lend support to Reeve and Shin's suggestion (2020) that autonomy‐supportive teaching practices, including adapting a matching vocal tone, can be taught. The softer approach shown after training suggests that trainees paid attention to the way they talk and that they aimed to communicate openness and interest to their pupils.

## LIMITATIONS AND FUTURE DIRECTIONS

To the best of our knowledge, this is the first study that explores the influence of teacher voice awareness training on motivational prosody production. Based on our and others' findings (Timmermans et al., 2011), we conclude that even short voice training programs can be effective in initiating change in vocal production. However, it should be noted that the current study did not compare the trainees' output with a control group that received no training, and future studies can build on the present findings by conducting well‐controlled intervention studies that record teachers within an actual classroom environment.

In addition, COVID‐19 restrictions made it necessary to record voices online, with little control over trainees' auditory recording setup (e.g. quality of microphone, distance from desktop/laptop). While it seems unlikely that all trainees systematically varied their recording position and equipment in pre‐ and post‐test sessions, any artefacts introduced through this lack of control would only have introduced noise, particularly to measures related to amplitude, that may have reduced effect sizes. Past research (e.g. Awan et al., 2024; Fraile & Godino‐Llorente, 2014) has shown that CPP measurements (here used as a measure of voice quality) are sensitive to noise in the signal. Specifically, Awan et al. (2024) compared different recording environments and showed that noisier environments lead to a reduction in CPP. In how far this could have reduced CPP effects here is unclear as SNR was better in session 1 than in the final session, yet CPP measures went up after training. Future studies should continue to monitor different voice quality indexes to estimate the usefulness of voice awareness training on these parameters. What also remains to be tested is how long‐lasting these effects are and whether the change in vocal production helps teachers in their day‐to‐day work beyond improving their vocal health. For example, it should be directly tested how effective the different voice patterns are when tackling typical classroom situations.

Linking the current work to the wider literature of the importance of self‐determination in the classroom (see review in Guay, 2022), the present results are promising in that they show that raising awareness around the benefits of supportive and encouraging voice usage, paired with practical tips on how to produce a supportive sounding voice, leads teachers to employ a softer voice for all materials tested. Whether this ultimately helps teachers to improve their classroom environment should be the focus of future studies. An additional avenue for further work links to past observations that children will not learn as effectively if spoken to in the ‘wrong’ voice (e.g. Kashinsky & Wiener, 1969), a finding particularly enhanced for socially disadvantaged children. The impact of vocal delivery in the classroom has also been noted by those working with neurodiverse children (Templeton, 2022), with authors arguing for softer tones when talking to neuro‐diverse children in particular.

As part of this future work, additional acoustic variables (e.g. harmonics‐to‐noise ratio, pitch slope, loudness) could be extracted to derive a more comprehensive picture of different motivational voices. Naturally, researchers will have to strike a balance between naturalistic and laboratory recording environments which will impact ability to extract and report features. The current results should be taken as a starting point to help devise research programs that directly feed into awareness training. Ultimately, hitting the right ‘tone’ will be as important for teaching content as it is when aiming to create learning environments in which children feel safe and supported.

## CONCLUSION

While expressions were not directly recorded in the classroom and no instructions had been given on which intentional category materials might fall into, the fact that vocal changes were observed between the first and last training sessions suggests the potential for voice awareness training in helping create a safe and positive learning environment as has been requested by the Department for Education (2015). There is now accumulating evidence that different voice patterns can affect listeners' behaviour (e.g. pressuring tones create rebellion; Weinstein et al., 2019), influence well‐being and connectedness (e.g. Weinstein et al., 2018) not only outside the classroom, but also within (Paulmann & Weinstein, 2023). It is tempting to fall back on firm and harsh communications, especially when speakers are stressed or students' behaviour challenging (Van Der Kaap‐Deeder et al., 2019). Moreover, classroom generated stress has been linked to shouting behaviour (Morton & Watson, 1998). Falcon et al. (2023) showed with live recordings in the classroom that children reacted to emotional intense voices with a reduction in performance, in line with work by Weinstein et al. (2019) that harsh voices can lead to defiance. If teachers were routinely equipped with the knowledge on the effects of soft and demanding voices, this could significantly enhance the classroom experience.

## AUTHOR CONTRIBUTIONS

**Silke Paulmann:** Conceptualization; investigation; methodology; funding acquisition; writing – original draft; writing – review and editing; formal analysis; project administration. **Netta Weinstein:** Conceptualization; methodology; writing – original draft; writing – review and editing; formal analysis; project administration.

## CONFLICT OF INTEREST STATEMENT

The authors have no conflict of interest to declare.

## Data Availability

The data that support the findings of this study will be made openly available upon publication.

## Footnotes

^1^
The study has received ethical approval through the Science and Health Subcommittee of the University of Essex.

^2^
Note that this age range is slightly larger than reported by others (e.g. Kovacic, 2005; Nusseck et al., 2021) who have studied teachers or trainees who were in their 20's. Roy et al. (2004), however, has also studied participants between the ages of 20 and 66 years.
